# Electrical impedance along connective tissue planes associated with acupuncture meridians

**DOI:** 10.1186/1472-6882-5-10

**Published:** 2005-05-09

**Authors:** Andrew C Ahn, Junru Wu, Gary J Badger, Richard Hammerschlag, Helene M Langevin

**Affiliations:** 1 Division for Research and Education in Complementary and Integrative Medical Therapies, Harvard Medical School, Boston, MA, USA; 2 Departments of Physics, University of Vermont, Burlington, VT, USA; 3 Department of Medical Biostatistics, University of Vermont, Burlington, VT, USA; 4 Research Department, Oregon College of Oriental Medicine, Portland OR, USA; 5 Department of Neurology, University of Vermont, Burlington, VT, USA

## Abstract

**Background:**

Acupuncture points and meridians are commonly believed to possess unique electrical properties. The experimental support for this claim is limited given the technical and methodological shortcomings of prior studies. Recent studies indicate a correspondence between acupuncture meridians and connective tissue planes. We hypothesized that segments of acupuncture meridians that are associated with loose connective tissue planes (between muscles or between muscle and bone) visible by ultrasound have greater electrical conductance (less electrical impedance) than non-meridian, parallel control segments.

**Methods:**

We used a four-electrode method to measure the electrical impedance along segments of the Pericardium and Spleen meridians and corresponding parallel control segments in 23 human subjects. Meridian segments were determined by palpation and proportional measurements. Connective tissue planes underlying those segments were imaged with an ultrasound scanner. Along each meridian segment, four gold-plated needles were inserted along a straight line and used as electrodes. A parallel series of four control needles were placed 0.8 cm medial to the meridian needles. For each set of four needles, a 3.3 kHz alternating (AC) constant amplitude current was introduced at three different amplitudes (20, 40, and 80 μAmps) to the outer two needles, while the voltage was measured between the inner two needles. Tissue impedance between the two inner needles was calculated based on Ohm's law (ratio of voltage to current intensity).

**Results:**

At the Pericardium location, mean tissue impedance was significantly lower at meridian segments (70.4 ± 5.7 Ω) compared with control segments (75.0 ± 5.9 Ω) (*p *= 0.0003). At the Spleen location, mean impedance for meridian (67.8 ± 6.8 Ω) and control segments (68.5 ± 7.5 Ω) were not significantly different (*p *= *0.70*).

**Conclusion:**

Tissue impedance was on average lower along the Pericardium meridian, but not along the Spleen meridian, compared with their respective controls. Ultrasound imaging of meridian and control segments suggested that contact of the needle with connective tissue may explain the decrease in electrical impedance noted at the Pericardium meridian. Further studies are needed to determine whether tissue impedance is lower in (1) connective tissue in general compared with muscle and (2) meridian-associated vs. non meridian-associated connective tissue.

## Background

In classic Chinese medicine theory, acupuncture meridians represent channels through which energy or "meridian *qi*" flows. Acupuncture points, traditionally located along these meridians, embody needling sites where the flow of *qi *may be affected. Acupuncture points and meridians are at the core of traditional acupuncture practice, yet anatomical and physiological explanations for these concepts remain elusive.

One widespread, yet controversial, explanation for acupuncture meridians involves electrical activity. In the acupuncture community, it is widely believed that acupuncture points and meridians are endowed with unique electrical properties. This constitutes the underlying assumption behind the use of electrical point locators commonly used in clinical practice and research. The experimental evidence in support of this practice, however, has been equivocal to date. Prior studies have reported that acupuncture points possess increased electrical conductivity compared to non-acupuncture points, and proposed that acupuncture meridians act as conduits for electrical current [1-16]. These studies, in general, were limited by small sample sizes, poor research design and procedural descriptions, and/or lack of rigorous statistical analyses. In addition, most studies used surface electrodes which may cause confounding by various factors including pressure, skin moisture, electrode contact and abrasion of the stratum corneum. For these reasons, the associations between acupuncture points or meridians and certain electrical properties have remained controversial.

Another proposed explanation for acupuncture points and meridians involves connective tissue. Recent studies have shown that acupuncture points exhibit a different biomechanical response to needling compared with non-acupuncture points [17], that this biomechanical response involves connective tissue [18,19], and that the network formed by acupuncture meridians may correspond to the body-wide network formed by connective tissue [20]. While the physiologic significance of this anatomical association is at present unclear, some researchers have proposed that the collagen content within connective tissue imparts electrical conductive properties [21,22]. These authors also suggested that connective tissue may act as the medium through which electrical communications travel within the acupuncture meridian network.

The goal of this study was to combine ultrasound evaluation and tissue impedance measurements to examine the electrical properties of connective tissue planes associated with meridians. We hypothesized that electrical impedance (which is inversely proportional to electrical conductivity) is lower along two acupuncture meridians associated with loose connective tissue planes (between muscles or between muscle and bone) visible by ultrasound, compared with non-meridians. In order to overcome the limitations associated with prior electrodermal studies, we used gold-plated acupuncture needles inserted into the tissues instead of surface electrodes and used a four-electrode technique with digital data acquisition to measure tissue electrical impedance. In this technique, four electrodes are placed in a straight line, a constant amplitude alternating (AC) current is passed between the two outer electrodes while voltage is measured between the two inner electrodes, and electrical impedance is calculated as the ratio of current to voltage amplitudes.

## Methods

### Setting

Twenty four subjects (18 female, 6 male) were recruited to participate in the study. Participants were recruited via flyers placed throughout the University of Vermont campus area. Subjects were excluded if they were under 18 years old, pregnant, used anticoagulation medications or had a history of bleeding disorder. One female subject withdrew part way through testing, leaving 23 subjects for analysis. Subjects' age was 39 ± 11.8 (mean ± SD) years. Demographic representation was: 18 non-Hispanic White, 2 Hispanic, 2 Native American and 1 Asian. Each subject was compensated for participation. The testing was performed in the General Clinical Research Center at the University of Vermont Medical Center from April to June 2004. The Institutional Review Board at the University of Vermont Medical Center approved the study methods and procedures.

### Instruments

**Figure 1:**
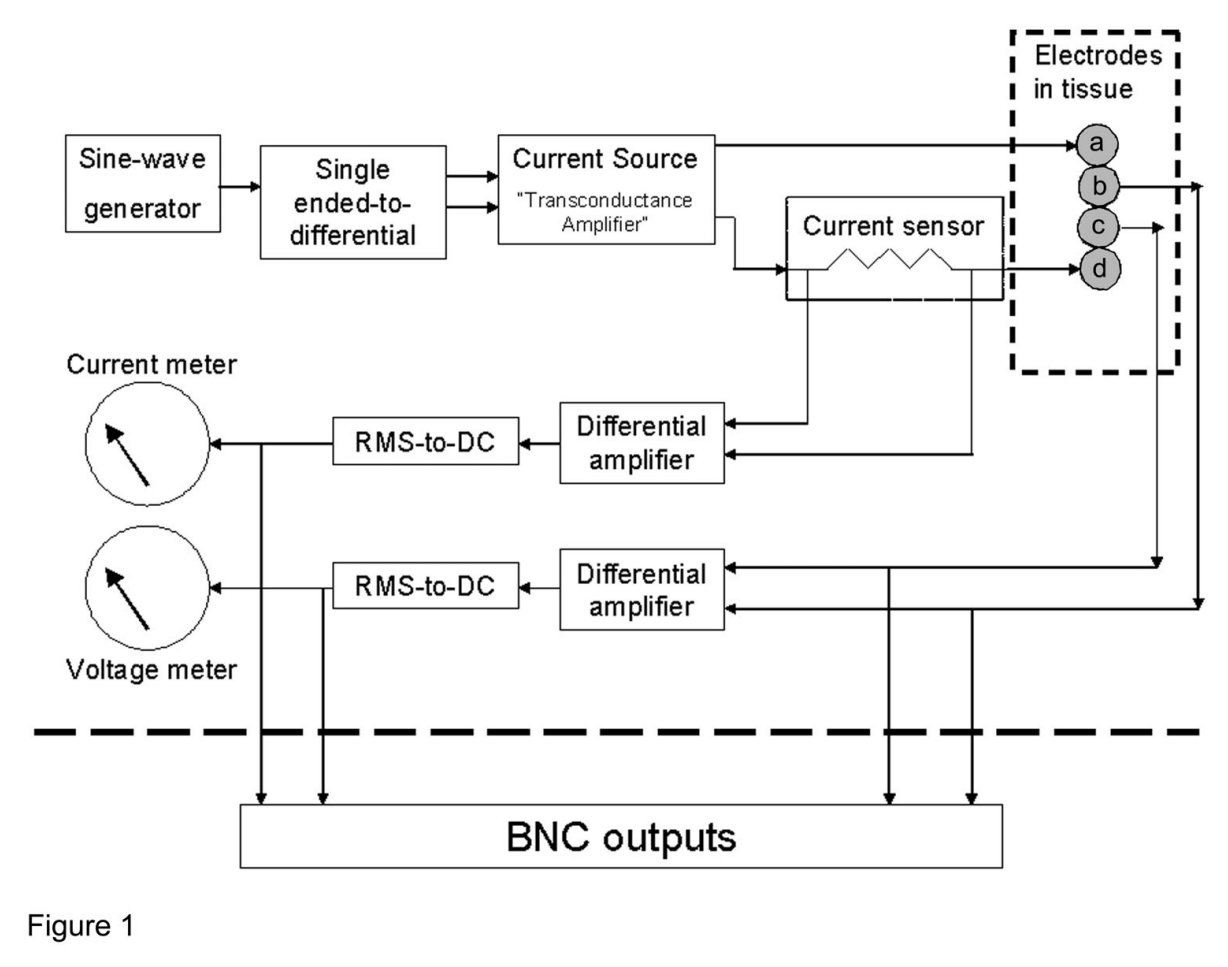
**Block diagram of impedance meter. **Through a rechargeable battery, a sine-wave alternating current is delivered to the outer two electrodes (a) and (d). A current sensor registers the amount of current delivered to the electrodes. The inner electrodes (b) and (c) are attached to the voltmeter which registers the electrical potential difference between them. The current and voltage readings may be recorded as time series through connections to a computer (BNC outputs).

An impedance meter based on the four-electrode technique was designed and fabricated specifically for this study. Prior to initiation of the study, rigorous evaluation of the instrument was performed *in vitro *and *in vivo*. The impedance meter outputs a regulated AC 3.3 kHz sinusoidal current of variable amplitude (20, 40, and 80 μA) between two outer electrodes (a) and (d) as shown in Figure 1. Voltage is measured by a voltmeter located between two inner electrodes (b) and (c) (Figure 1). The current used is alternating (AC) as opposed to direct (DC) current to avoid saturating of the electrode surfaces as a result of mobile ion build up (electrolysis). The four-electrode method is superior to a two-electrode method because it minimizes error caused by the contact impedance between electrodes and tissue. The instrument was calibrated by measuring known precision resistors with a commercial Wheatstone bridge; errors were less than 1%. Furthermore, preliminary testing of voltage measurements using gold-plated acupuncture needles in 0.9% NaCl solution indicated stable readings over a 15 minute period and did not change when the polarity of the electrodes was reversed. Standard stainless steel acupuncture needles also were tested but demonstrated substantial voltage fluctuations due to reaction of the metal with the ionic solution. An AC frequency of 3.3 kHz was low enough to minimize ambient electrical interference occurring at higher frequencies and high enough to avoid electrode saturation occurring at lower frequencies.

### Procedure

**Figure 2:**
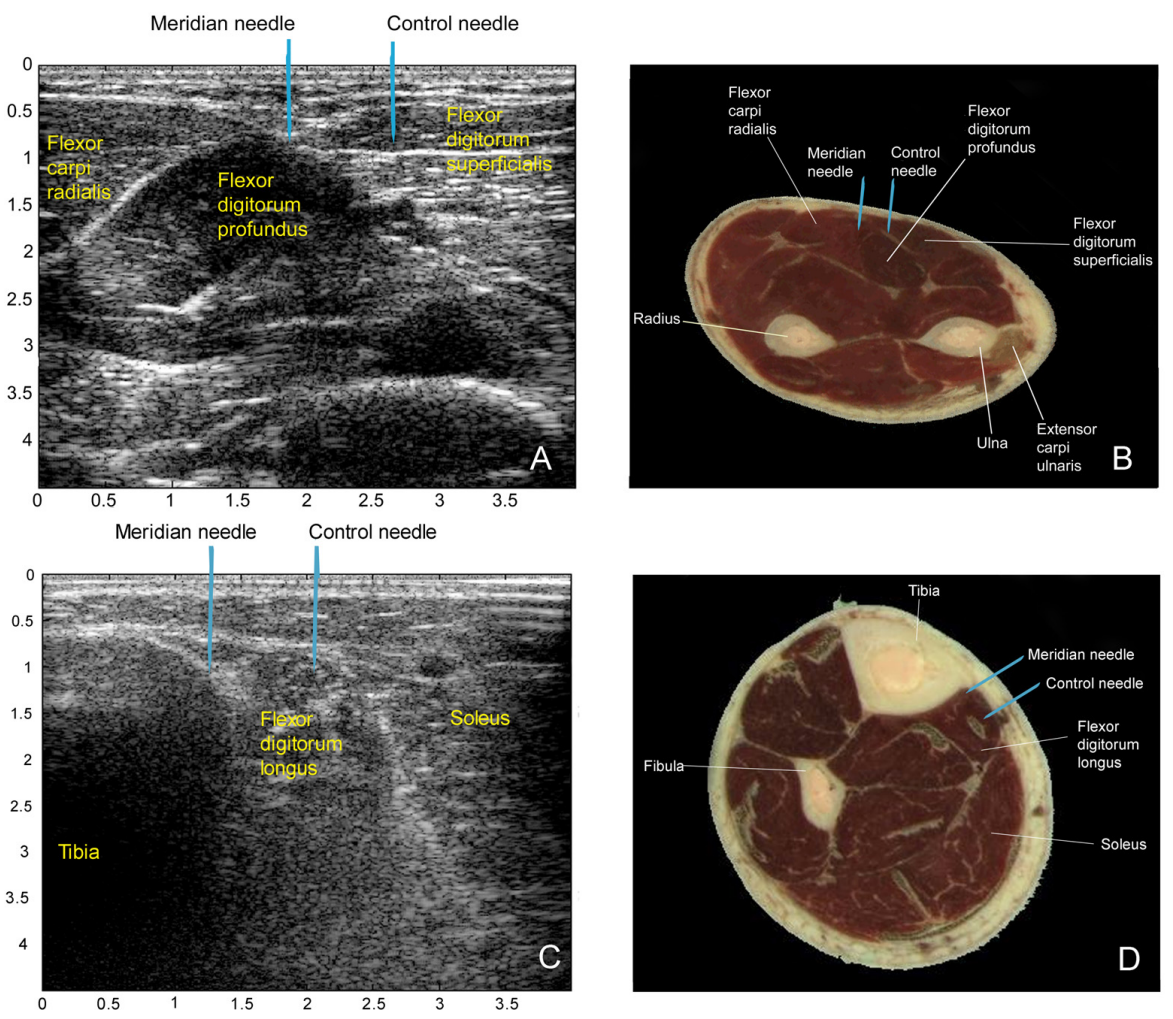
**Depiction of the Spleen and Pericardium meridians in relation to surface and sub-surface anatomical landmarks. **2 A, B: Cross sectional images of the right forearm as shown through ultrasound and gross anatomical cross section (obtained from the Visual Human Database). The Pericardium segment is located between the flexor carpi radialis and flexor digitorum superficialis muscles. 2 C, D: Cross sectional images of the right leg as shown through ultrasound and cadaveric cross section. The Spleen segment is located between the medial crest of the tibia and the flexor digitorum longus muscle. Blue arrows point to approximate sites where meridian and control needles were inserted.

Two different segments on the skin surface located along acupuncture meridians were chosen for measurement. These segments corresponded to portions of the Pericardium and Spleen meridians [23,24] and will herein be referred to as "Pericardium meridian segment" and "Spleen meridian segment" respectively. The segments were chosen because they were straight, located on flat skin surfaces and easily accessible for ultrasound scanning. The Pericardium segment was located between the flexor carpi radialis and flexor digitorum superficialis muscles, whereas the Spleen segment was located between the medial crest of the tibia and the flexor digitorum longus muscle. The proximal end of the Pericardium meridian segment was one *cun *(a unit of proportional measurements used in acupuncture practice [24]) distal to the elbow crease and the distal end of the Spleen meridian segment was four *cun *proximal to the medial malleolus. The location of each meridian and control segment was drawn on the surface of the skin with a marker. Control segments were located 0.8 cm medial and parallel to each meridian segment. Representative ultrasound images for the meridian and control segments are displayed in Figure 2. Ultrasound images were obtained using a GE (Vingmed) System Five (Oslo, Norway) scanner with a 10 MHz linear array probe and an imaging depth of 45 mm. Images were obtained transversely to the meridian and control segments. The right arm and right leg were used for testing. In certain cases where scarring, cysts, or skin inflammation precluded testing on the right side, the left side was used instead for both ultrasound imaging and needling.

**Figure 3:**
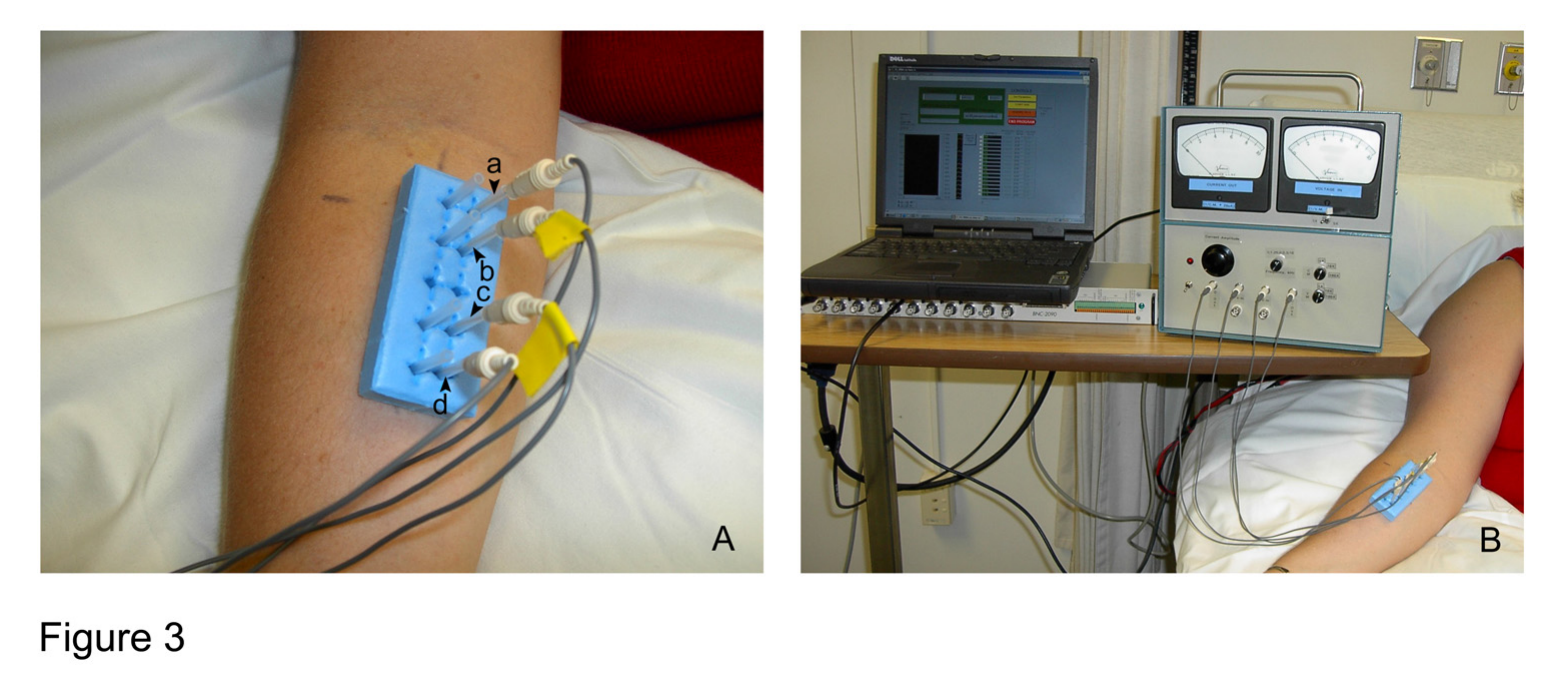
**Experimental setup. **3A- Holder placed on Pericardium meridian and control skin segments. Guide tubes indicate the location of needles on both segments. In this image, needles (a), (b), (c), and (d) are inserted along the control segment. Current is passed between electrodes (a) and (d) and voltage is measured between (b) and (c). 3B- Tissue impedance meter connected to laptop computer and to needle electrodes.

After cleaning the skin with alcohol, a sterile adhesive holder (Suture aid booties, Sterion Incorporated, Ham Lake, MN) was placed on the skin at each location. Each holder held sterile acupuncture needle guide tubes (four for the meridian needles and four for the control needles) that each had been cut to a length of 33 mm (Figure 3A). The four meridian guide tubes were placed over the meridian segment, with the proximal guide tube located at the proximal end of the segment. Within meridian and control segments, the guide tubes were 13 mm apart, with the exception of the two inner guide tubes where the interval distance was 25 mm. This created a scaffold for the needles to maintain consistent (1) inter-needle distance within a given segment, (2) inter-segment distance between meridian and control segments and (3) needle insertion depth.

For each segment, the order of testing (meridian vs. control) was randomized. After randomization, four gold-plated needles (Viva 0.25 mm diameter, 40 mm length, Helio Medical Supplies, Inc., San Jose, CA) were connected to the impedance meter device via electrical cables (Teca, Oxford Instruments Medical, Hawthorne, NY). The handle of each acupuncture needle was inserted 10 mm into the female end of the connector cable, while the male end was inserted into the impedance meter device (Figure 3). The gold plated acupuncture needles thus acted as electrodes. Four needles were inserted through each of the four guide tubes overlying either a meridian or control segment and then into the skin and underlying tissues. Each needle was inserted until the hub of the connector contacted the end of the guide tube. This ensured that all needles were inserted to the same depth of 10 mm. In all cases, electrodes (a) and (d) were connected to the two output electrodes of the constant current source and electrode (a) was inserted into the most proximal needling site. A 3.3 kHz AC current was then introduced at three different amplitudes (20, 40, and 80 μA) to the outer two needles while the voltage was measured between the inner two needles. Measurements were made twice for two to five seconds at each current amplitude. The voltage was read on an analogue voltmeter as well as stored as a computer digital time series using LabView 5.1 software and data acquisition board (National Instruments Corporation, Austin, TX). During the testing, subjects laid supine on a standard hospital bed with the head elevated at approximately 60 degrees above horizontal. Tissue impedance was determined by taking the slope of the linear regression of voltage as a function of current intensity using least squares methodology.

### Statistical analysis

Two-factor repeated measures analysis of variance was used to test for differences in mean tissue impedance across experimental conditions. The repeated factors represented segment (meridian vs. control), and location (Pericardium vs. Spleen). If there was evidence of an interaction between the two factors (i.e. segment differences were determined to be location dependent), simple effects within each location were examined based on an F-test corresponding to the appropriate contrast. Statistical analyses were performed using SAS statistical software (SAS Version 8, SAS Institute, Cary, NC).

## Results

**Figure 4:**
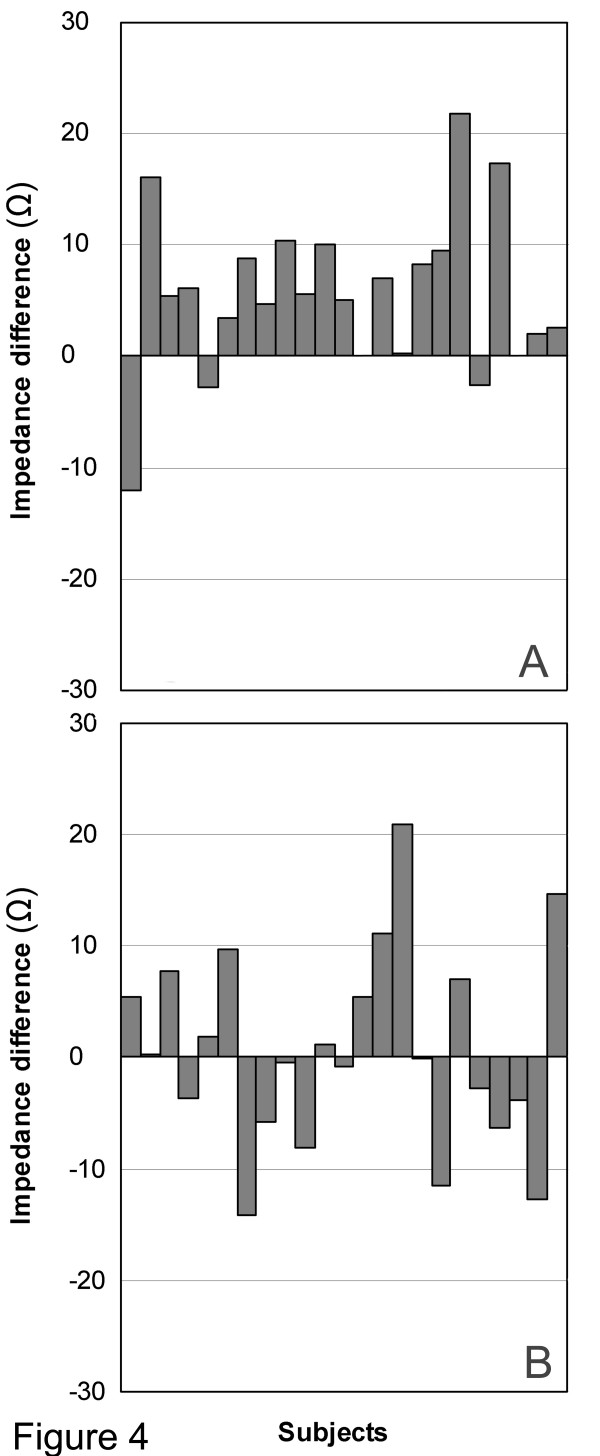
**Tissue impedance measurements for individual subjects. **The difference in impedance between control and meridian segments (Impedance difference) is shown for the Pericardium (A) and Spleen (B) meridian location.

**Figure 5:**
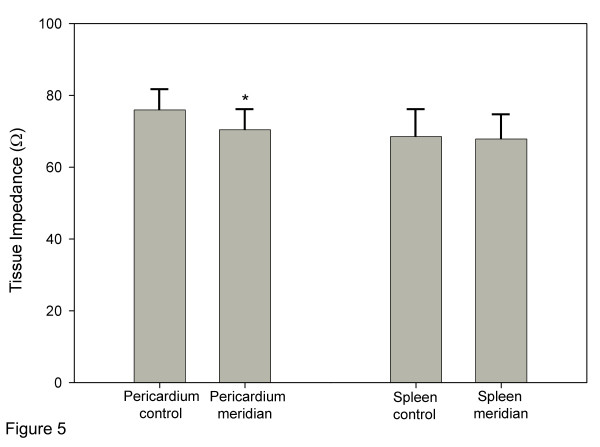
**Mean tissue impedance at Pericardium and Spleen meridian segments and at corresponding control segments. **Bar graphs represent mean ± SE. * indicates p < 0.01.

Differences in tissue impedance between control and meridian segments for individual subjects are shown in Figure 4. Overall, there was a significant difference in mean tissue impedance between meridian and control segments (Repeated Measures ANOVA, *main effect p *= 0.004). There was also near-significant evidence suggesting that the difference was location-specific (Repeated Measures ANOVA, *interaction p *= 0.07). When comparisons were performed within each location, difference in mean tissue impedance at the Pericardium location were highly significant (*p *= 0.0003) (Figure 5). Tissue impedance was 70.4 ± 5.7 Ω (mean ± SE) for the Pericardium meridian segment and 75.0 ± 5.9 Ω for the Pericardium control segment. Analyses performed within the Spleen location did not result in significant differences between the two segments (*p *= *0.70*) (Figure 5). Tissue impedance was 67.8 ± 6.8 Ω and 68.5 ± 7.5 Ω for Spleen meridian and control segments respectively.

## Discussion

We found that tissue impedance was lower along the Pericardium meridian (compared with control) but not along the Spleen meridian. Several factors may have contributed to this observed difference. First, although both Pericardium and Spleen meridian segments were associated with loose connective tissue planes (between muscles or between muscle and bone), the Pericardium meridian-associated connective tissue was in general more clearly defined in the ultrasound images than the Spleen meridian-associated connective tissue. Second, in clinical settings, the Spleen channel may need to be needled deeper than the Pericardium channel [25-27] suggesting that the 1 cm needle penetration at the Spleen segment may not have been sufficient. Third, the proximity of control segments to other (non-meridian/meridian) connective tissue planes may also have influenced our results. The Pericardium control needles were inserted into the flexor digitorum superficialis muscle which has a relatively wide transverse width (Figure 2A) and therefore the Pericardium control needles were inserted into the belly of that muscle in nearly all subjects. In contrast, because of the variable width of the flexor digitorum longus and its orientation perpendicular to the skin surface, the Spleen control segment was often close to the connective tissue plane separating this muscle from the soleus (Figure 2C). Therefore in some subjects, the Spleen control needle may have penetrated as much connective tissue as the Spleen meridian needles. Indeed in the ten subjects for which both medial and lateral edges of the flexor digitorum longus could be clearly delineated on the ultrasound images, greater flexor digitorum longus muscle width was positively correlated with greater tissue impedance in Spleen control relative to meridian segments (r = 0.60). In this regard, it is interesting to note that there is some variability among major acupuncture texts about the respective locations of the Spleen, Kidney and Liver meridians in this portion of the leg [23,25,27], all of these meridians running longitudinally medial to the medial edge of the tibia. Thus the connective tissue plane separating the flexor digitorum longus and soleus may in fact represent the Kidney or Liver meridian. On the other hand, this connective tissue plane may not represent any acupuncture meridian. Further studies are needed to (1) test segments located over inter-muscular connective tissue planes (whether meridian-associated or not), compared with segments located over muscle to determine whether connective tissue in general has decreased impedance compared with muscle and (2) test segments located over meridian-associated connective tissue vs. non-meridian associated connective tissue to determine whether meridians have decreased impedance compared with non meridian-associated connective tissue. Selection of areas where connective tissue planes are spaced as far apart as possible (such as the thigh and the upper arm) would be advantageous to test these hypotheses.

Compared to previous studies examining the electrical properties of acupuncture points and meridians, our measurement methods offer several advantages. First, we used needles to overcome the variability associated with surface electrodes. By directly accessing the tissue, the needles bypass potential confounders such as pressure, sweat, skin abrasions, and variable surface topography, among other factors. Second, we utilized a four-electrode method to perform our tissue impedance measurements. To date, most investigations of electrical impedance of acupuncture points or meridians have used a two-electrode method, i.e., the two electrodes used to introduce an electrical current were also connected to a voltmeter to measure the voltage between the two electrodes [1-4,6-8,10,11,14,15]. This method can cause significant fluctuation of voltage between the two electrodes due to variable contact impedance between electrodes and tissue. The four electrode technique used in this study is considered the standard in biophysical sciences and is widely employed to measure electrical conductance/impedance of biological tissue [28-37]. This method minimizes error due to fluctuation in voltage and electrode contact impedance. Three prior studies have evaluated the bioelectrical properties of acupuncture meridians using a four-electrode technique *in vivo *[38,39] and using an *in vitro *gel model [40]. Both *in vivo *studies reported finding lower electrical impedance along acupuncture meridians. However, in one study [39] the four electrodes were not all placed along a straight line, making it difficult to relate that study's results to our findings, and in the other the results were not analyzed statistically [38].

Since the investigator was not blinded to which segment corresponded to the meridian and which to the control, guarding against potential sources of experimental bias during needle placement and data collection was an important aspect of this study. Use of (1) adhesive scaffolding and needle guide tubes (ensuring that all needles were equidistant and inserted to the same depth) and (2) computerized data collection (analogue data was only collected as a backup for the digital files) ensured that minimal opportunity was present for bias to influence the experimental results.

This study has several limitations. Because we used uninsulated needles, our measurements reflected the total effective impedance between the two inner electrodes and therefore we could not resolve components of this tissue impedance contributed by individual tissue layers. In addition, the study of electrical properties of biological tissues is extremely complex, and the presence of tissue anisotropy, possible biological semiconductor properties (lending to internal charge storage between adjacent biological structures), and tissue heterogeneity precludes attributing the observed differences in electrical impedance to any particular molecule or structure. These complexities will present challenges for future studies in delineating exact causes for any possible electrical communication within the acupuncture network. Given these complexities, however, it is remarkable that the impedance values of the Pericardium segment were consistently lower than that of its control. This study will provide initial groundwork for future, more extensive investigations.

## Conclusion

In summary, tissue impedance was lower along the Pericardium meridian (compared with control) but not along the Spleen meridian. Ultrasound imaging of meridian and control segments suggest that (1) lack of difference at the Spleen location may have been due to both control and meridian needles penetrating connective tissue and (2) tissue impedance may be influenced by needle penetration of connective tissue, whether meridian-associated or not.

## Competing interests

The author(s) declare that they have no competing interests.

## Authors' contributions

AA – Participated in testing of human subjects and wrote the manuscript.

JW – Designed and supervised *in vitro *testing and calibration of the tissue impedance instrument.

GJB – Conducted statistical analysis and assisted in writing of the manuscript.

RH – Participated in designing the study and *in vitro *testing of tissue impedance instrument and edited the manuscript.

HML – Supervised the design and coordination of the study, testing, and manuscript preparation.

## Pre-publication history

The pre-publication history for this paper can be accessed here:



## References

[B1] Chen KG (1996). Electrical properties of meridians. IEEE Eng Med Biol Mag.

[B2] Hyvarinen J, Karlsson M (1977). Low-resistance skin points that may coincide with acupuncture loci. Med Biol.

[B3] Nakatani Y, Yamashita K (1977). Ryodoraku Acupuncture.

[B4] Nakatani Y (1956). Skin electric resistance and ryodoraku. J Autonomic Nerve.

[B5] Niboyet J (1958). Nouvelle constatations sur les proprietes electriques des ponts Chinois. Bull Soc Acup.

[B6] Reichmanis M, Marino AA, Becker RO (1976). DC skin conductance variation at acupuncture loci. Am J Chin Med.

[B7] Reichmanis M, Marino AA, Becker RO (1975). Electrical correlates of acupuncture points. IEEE Trans Biomed Eng.

[B8] Becker RO, Reichmanis M, Marino AA, Spadaro JA (1976). Electrophysiological correlates of acupuncture points and meridians.. Psychoenergetic Systems.

[B9] Becker RO, Selden G (1985). The body electric : electromagnetism and the foundation of life.

[B10] Terral C, Rabischong P (1997). A scientific basis for acupuncture?. The Journal of Alternative and Complementary Medicine.

[B11] Voll R (1977). Nosodenanwendung in Diagnostik und therapie.

[B12] Zhu Z (1981). Research advances in the electrical specificity of meridians and acupuncture points. American Journal of Acupuncture.

[B13] Brown ML, Ulett GA, Stern JA (1974). Acupuncture loci: techniques for location. Am J Chin Med.

[B14] Reichmanis M, Marino AA, Becker RO (1977). Laplace plane analysis of transient impedance between acupuncture points Li-4 and Li-12. IEEE Trans Biomed Eng.

[B15] Nakatani Y (1956). An aspect of the study of Ryodoraku. Clinic of Chinese Medicine.

[B16] Niboyet JEH, Bourdiol RJ, Regard PG (1970). Traite d'acupuncture. Traité d'acupuncture.

[B17] Langevin HM, Churchill DL, Fox JR, Badger GJ, Garra BS (2001). Biomechanical response to acupuncture needling in humans. J Appl Physiol.

[B18] Langevin HM, Churchill DL, Cipolla MJ (2001). Mechanical signaling through connective tissue: A mechanism for the therapeutic effect of acupuncture. FASEB J.

[B19] Langevin HM, Churchill DL, Wu J, Badger GJ, Yandow JA, Fox JR, Krag MH (2002). Evidence of connective tissue involvement in acupuncture. FASEB J.

[B20] Langevin HM, Yandow JA (2002). Relationship of acupuncture points and meridians to connective tissue planes. Anat Rec (New Anat).

[B21] Ho MW, Knight DP (1998). The acupuncture system and the liquid crystalline collagen fibers of the connective tissues. Am J Chin Med.

[B22] Oschman JL (2000). Energy medicine : the scientific basis.

[B23] Deadman P, Al-Khafaji M, Baker K (1998). A Manual of Acupuncture.

[B24] Cheng XM (1995). Acupuncture Reference Book.

[B25] Cheng H, Teng L, Cheng H and Teng L (1987). Chinese acupuncture and moxibustion. Chapter 14: Acupuncture Techniques.

[B26] Beijing, Shanghai and Nanjing Colleges of Traditional Chinese Medicine (1980). Essentials of Chinese Acupuncture..

[B27] O'Connor J, Bensky D, Shanghai Zhong yi xue yuan. (1981). Acupuncture : a comprehensive text.

[B28] Lukaski HC (2003). Regional bioelectrical impedance analysis: applications in health and medicine. Acta Diabetol.

[B29] Salazar Y, Bragos R, Casas O, Cinca J, Rosell J (2004). Transmural versus nontransmural in situ electrical impedance spectrum for healthy, ischemic, and healed myocardium. IEEE Trans Biomed Eng.

[B30] Cinca J, Warren M, Carreno A, Tresanchez M, Armadans L, Gomez P, Soler-Soler J (1997). Changes in myocardial electrical impedance induced by coronary artery occlusion in pigs with and without preconditioning: correlation with local ST-segment potential and ventricular arrhythmias. Circulation.

[B31] Ellenby MI, Small KW, Wells RM, Hoyt DJ, Lowe JE (1987). On-line detection of reversible myocardial ischemic injury by measurement of myocardial electrical impedance. Ann Thorac Surg.

[B32] Fallert MA, Mirotznik MS, Downing SW, Savage EB, Foster KR, Josephson ME, Bogen DK (1993). Myocardial electrical impedance mapping of ischemic sheep hearts and healing aneurysms. Circulation.

[B33] Steendijk P, van der Velde ET, Baan J (1994). Dependence of anisotropic myocardial electrical resistivity on cardiac phase and excitation frequency. Basic Res Cardiol.

[B34] Tsai JZ, Cao H, Tungjitkusolmun S, Woo EJ, Vorperian VR, Webster JG (2000). Dependence of apparent resistance of four-electrode probes on insertion depth. IEEE Trans Biomed Eng.

[B35] Tsai JZ, Will JA, Hubbard-Van Stelle S, Cao H, Tungjitkusolmun S, Choy YB, Haemmerich D, Vorperian VR, Webster JG (2002). Error analysis of tissue resistivity measurement. IEEE Trans Biomed Eng.

[B36] Tsai JZ, Will JA, Hubbard-Van Stelle S, Cao H, Tungjitkusolmun S, Choy YB, Haemmerich D, Vorperian VR, Webster JG (2002). In-vivo measurement of swine myocardial resistivity. IEEE Trans Biomed Eng.

[B37] Haemmerich D, Staelin ST, Tsai JZ, Tungjitkusolmun S, Mahvi DM, Webster JG (2003). In vivo electrical conductivity of hepatic tumours. Physiol Meas.

[B38] Yang W, Chang R Investigation of lower resistance meridian
I. Method of investigation. Peking University Academic Journal.

[B39] Zhang W, Xu R, Zhu Z (1999). The influence of acupuncture on the impedance measured by four electrodes on meridians. Acupunct Electrother Res.

[B40] Zhang W, Zhuang F, Tian Y, Li H (2001). [A simulating study of biophysical features along meridians on a gel model]. Sheng Wu Yi Xue Gong Cheng Xue Za Zhi.

